# Next-generation sequencing-based genomic profiling of advanced soft tissue and bone sarcomas

**DOI:** 10.3389/fonc.2025.1627452

**Published:** 2025-10-03

**Authors:** Yasemin Gündoğdu, Elif Şenocak Taşçı, Leyla Özer, Can Boynukara, Recep Çeçen, Arda Ulaş Mutlu, İbrahim Yıldız

**Affiliations:** ^1^ Department of Internal Medicine, Acıbadem MAA University, Istanbul, Türkiye; ^2^ Department of Medical Oncology, Acıbadem MAA University, Istanbul, Türkiye; ^3^ Department of Medical Oncology, Memorial Hospital, Istanbul, Türkiye; ^4^ Department of Internal Medicine, Faculty of Medicine, Istanbul University Cerrahpaşa, Istanbul, Türkiye; ^5^ Department of General Surgery, Acıbadem MAA University, Istanbul, Türkiye

**Keywords:** sarcoma, soft tissue, next-generation sequencing, molecular, targeted therapy

## Abstract

**Background:**

Sarcomas are rare mesenchymal tumors classified into soft tissue (STS) and bone sarcomas. Despite advances in treatment, the 5-year survival rate for metastatic disease remains low. There is still limited evidence regarding the use of next-generation sequencing (NGS).

**Aim:**

To identify targetable genomic alterations that may play a crucial role in sarcoma treatment where therapeutic options are limited.

**Study design:**

**Methods:** We conducted a retrospective; multicenter analysis of 81 patients diagnosed with STS and bone sarcomas who underwent NGS at Acıbadem Health Group Hospitals to investigate their mutation profiles and explore potential targeted therapies.

**Results:**

Genomic profiling using four different NGS kits identified a total of 223 genomic alterations across the cohort. Genomic alterations were detectable in 90.1% of patients, with the most common types being copy number amplifications (26.9%) and deletions (24.7%). In addition, actionable mutations were identified in 22.2% of patients, rendering them eligible for FDA-approved targeted therapies. The most common alterations were found in *TP53* (38%), *RB1* (22%), and *CDKN2A* (14%) genes. Among the 79 patients with available microsatellite status data, all were microsatellite stable.

**Conclusion:**

The high proportion of patients eligible for targeted therapies identified underscores the critical need to integrate NGS-derived genetic insights into clinical practice to improve survival rates and treatment outcomes through more tailored therapeutic approaches for each individual. NGS also led to a reclassification of diagnosis in four patients, demonstrating its utility not only in therapeutic decision-making but also as a powerful diagnostic tool.

## Introduction

Sarcomas, a rare and heterogeneous group of tumors originating from mesenchymal cells, represent approximately 1% of adult malignancies and are broadly categorized into soft tissue sarcomas (STS) and bone sarcomas (1). With over 100 histological subtypes, these tumors exhibit distinct molecular and genetic alterations that significantly influence their biology, progression, and response to treatment (2). Despite advancements in treatment modalities, such as novel chemotherapeutics, immunotherapy, targeted therapies, and radiotherapy, the prognosis for sarcoma patients remains poor. Metastasis detection during follow-up after diagnosis remains high, with rates reported between 20% and 50% in various studies (3, 4).

Sarcomas can also be classified based on their genomic complexity into two major groups: simple karyotype (associated with translocations or specific activating mutations) and complex karyotype (marked by genomic heterogeneity and instability) (5). The identification and classification of sarcomas with complex karyotypes using conventional techniques, which often exhibit non-specific genomic alterations, present significant challenges (1). These complexities, coupled with the rarity of sarcomas, underscore the need for advanced tools to enhance diagnostic accuracy and optimize treatment strategies (6).

In recent years, the integration of comprehensive genomic profiling into clinical practice has begun to reshape standard care protocols, providing more personalized and precise therapeutic options (7). Among these innovations, next-generation sequencing (NGS) technologies have emerged as transformative tools in the diagnostic, prognostic, and therapeutic management of sarcomas. The 5th edition of the WHO classification of soft tissue and bone sarcomas, updated in 2020, highlights the significance of genetic mutations identified through NGS (2). NGS, with its advanced DNA and RNA sequencing capabilities, enables the simultaneous identification of multiple fusion mutations and previously unknown genetic alterations (8, 9). By offering a more detailed understanding of the genomic landscape of sarcomas, NGS facilitates targeted therapies tailored to specific mutational profiles (10).

However, despite its ability to identify numerous mutations through high-throughput sequencing, the clinical translation of these findings remains limited, with lower-than-expected rates of direct therapeutic benefit (11). Furthermore, tumors in patients receiving targeted therapies often develop resistance over time. In this context, NGS is a vital tool for uncovering resistance mechanisms and helping clinicians choose alternative treatment strategies, thereby advancing precision oncology in sarcomas.

In this study, we aim to investigate the genomic profiles of patients diagnosed with soft tissue and bone sarcomas using a comprehensive NGS-based approach and evaluate the potential impact of these findings in the management of sarcoma. As a referral center specialized in sarcoma treatment, we also seek to assess the role of NGS in improving diagnosis and treatment outcomes within our patient population.

## Materials and methods

### Study design and patient selection

In this multi-center, cross-sectional retrospective study (IRB number: 2024-4/150) patients diagnosed with soft tissue or bone sarcoma between 2017 and 2023 were analyzed. The study cohort included 81 adult patients diagnosed with STS (n=61) or bone sarcoma (n=20). Patients with gastrointestinal stromal tumors (GIST) and Kaposi sarcoma were excluded, as these tumors have distinct molecular characteristics and treatment approaches. Demographic and clinicopathological data were retrieved from electronic health records. All patients underwent either a biopsy or surgical resection, followed by standardized pathological examinations conducted by a team of sarcoma-specialized pathologists. The pathology specimens were consulted by the same specialized team if the patient is foreign.

### Comprehensive molecular profiling

All participants underwent comprehensive molecular profiling at various points during their diagnosis or treatment using NGS tests (Foundation Medicine, Inc. [FoundationOne], Tempus, OncoDEEP (OncoDNA SA) and MI profile (Caris Inc.)). All the tests were performed in CLIA- regulated laboratories. Genomic alterations, such as insertions/deletions, copy number variations and structural rearrangements were recorded. Tumor mutation burden (TMB, an estimate of the number of mutations per megabase) and microsatellite instability (MSI) status were also documented when available. Variants of unknown significance were not included in the analysis. All sequencing was performed on tumor tissue without matched normal controls. As a result, the ability to definitively distinguish somatic from germline variants was limited. In tumor-only NGS analyses, variants with a variant allele frequency (VAF) greater than 50% were considered suspicious for possible germline origin. In addition, pathogenic variants occurring in well-known hereditary cancer predisposition genes (such as BRCA1/2, TP53, ATM, CHEK2) were also reviewed for potential germline significance. In such cases, or when a hereditary cancer syndrome was clinically suspected, confirmatory germline testing was subsequently performed using validated germline assays. Germline mutations were confirmed in two patients (BLM, TP53, ATM), followed by genetic counseling and family risk assessment. Actionable alterations were initially determined based on clinical annotations provided by the respective NGS platforms. Because most platforms used in this study are aligned with or directly integrated into the OncoKB evidence-level classification system, all actionable mutations were retrospectively reclassified according to OncoKB criteria. This framework incorporates FDA approval status, clinical guideline support, and the strength of supporting clinical or biological evidence. The NGS platforms used in this study differed in terms of gene content, sequencing depth, and variant calling pipelines. TMB and MSI values were interpreted using platform-specific thresholds and algorithms as provided in certified clinical reports. Given the differences in analytical design across platforms, direct standardization was not feasible.

### Ethics

The study was conducted in accordance with the Declaration of Helsinki and approved by the Ethics Committee of Acıbadem MAA University (approval number: 2024-4/150, date: 14.03.2024). All methods and protocols were performed in accordance with the relevant guidelines and regulations.

## Results

### Patient characteristics

The study included 81 patients who underwent treatment for STS and bone sarcoma. The median age of patients was 45.2 years at diagnosis (range: 18-80) and there was slight female predominance (51.8%, n=42). Among these patients, 61 (75.3%) had STS while 20 (24.7%) were diagnosed with bone sarcoma. All patients had metastatic disease prior to performing NGS.

Undifferentiated pleomorphic sarcoma (UPS) was the most common STS subtype (22.7%), followed by leiomyosarcoma (16%) and synovial sarcoma (11.1%). The clinicopathological data of the patients is given in **Table 1**. The most frequently observed bone sarcomas were Ewing’s sarcoma (13.6%) and osteosarcoma (7.4%).

**Table 1:** Clinicopathological data of included patients (n=81).

	No (%)	No of Genomic Alterations	Genomic Alterations per Patient (range)	No of Patients with Targetable Alterations
Mean Age at diagnosis	45.2			
Gender				
Male	39 (48.2)			
Female	42 (51.8)			
Diagnosis				
Undifferentiated Pleomorphic Sarcoma	22 (22.7)	68	3.08 (0-9)	4
Leiomyosarcoma	16 (19.8)	39	2.44 (0-9)	3
Ewing Sarcoma	11 (13.6)	32	2.91 (0-6)	0
Synovial Sarcoma	9 (11.1)	19	2.1 (0-8)	3
Rhabdomyosarcoma	7 (8.6)	21	3 (2-6)	2
Osteosarcoma	6 (7.4)	17	2.82 (1-6)	2
Liposarcoma	3 (3.7)	16	5.32 (4-6)	3
Chondrosarcoma	2 (2.5)	5	2.5 (2-3)	0
Clear Cell Sarcoma	1 (1.2)	1	1	0
Desmoid Type Fibromatosis	1 (1.2)	1	1	0
Fibrosarcoma	1 (1.2)	1	1	0
Follicular Dendritic Cell Sarcoma	1 (1.2)	1	1	1
Spindle Cell Sarcoma	1 (1.2)	2	2	0
Total, mean (range)	81 (100)	223	2.74 (0-9)	18

### Genomic alterations and instability

**Figure 1:**
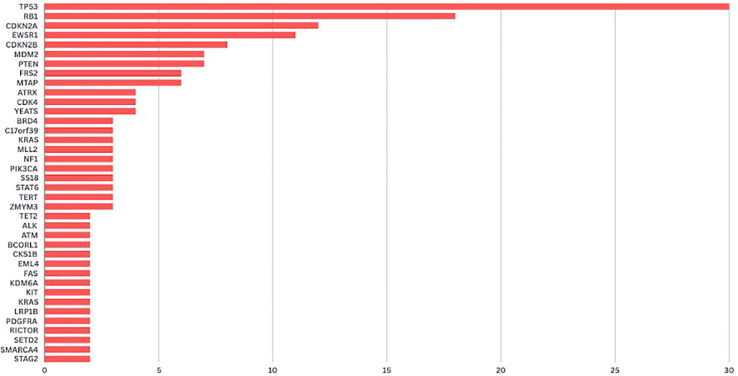
Number of genomic alterations detected in patients with sarcoma.

The four commercial NGS kits used were Tempus (Tempus Labs Inc.)(n:48), FoundationOne (Foundation Medicine Inc.)(n:24), OncoDEEP (OncoDNA SA)(n:6) and MI profile (Caris Inc.)(n:3). A total of 223 genomic alterations were identified across the cohort, with an average of 2.74 alterations per patient. Notably, at least one type of genomic alteration was present in 90.1% (n=73) of tumors. The remaining 8 did not possess any significant mutations according to the reports. The most frequently mutated genes were TP53 (38%, n=31), RB1 (22%, n=18) and CDKN2A (14%, n=12) followed by EWSR1 (13%, n= 11), CDKN2B (9%, n=8), MDM2 (8%, n= 7), PTEN (8%, n= 7), FRS2 (7%, n= 6) and MTAP (7%, n= 6) (**Figure 1**).

Upon functional analysis of genomic alterations, potentially targetable changes were identified in several key pathways. In the genomic stability regulation pathway, alterations were detected in TP53 (38%) and MDM2 (8%). In the cell cycle regulation pathway, mutations were observed in RB1 (22%), CDKN2A (14%), CDKN2B (9%), CDK4 (5%), and CDKN2A/B (1%). In the DNA repair pathway, alterations were found in RAD (1%). In the phosphoinositide-3 kinase (PI3K) pathway, mutations were identified in PTEN (8%), PIK3CA (4%), mTOR (1%), and RICTOR (2%). In the receptor tyrosine kinase pathway, alterations were detected in ALK (2%) and FGFR (4%).

**Figure 2:**
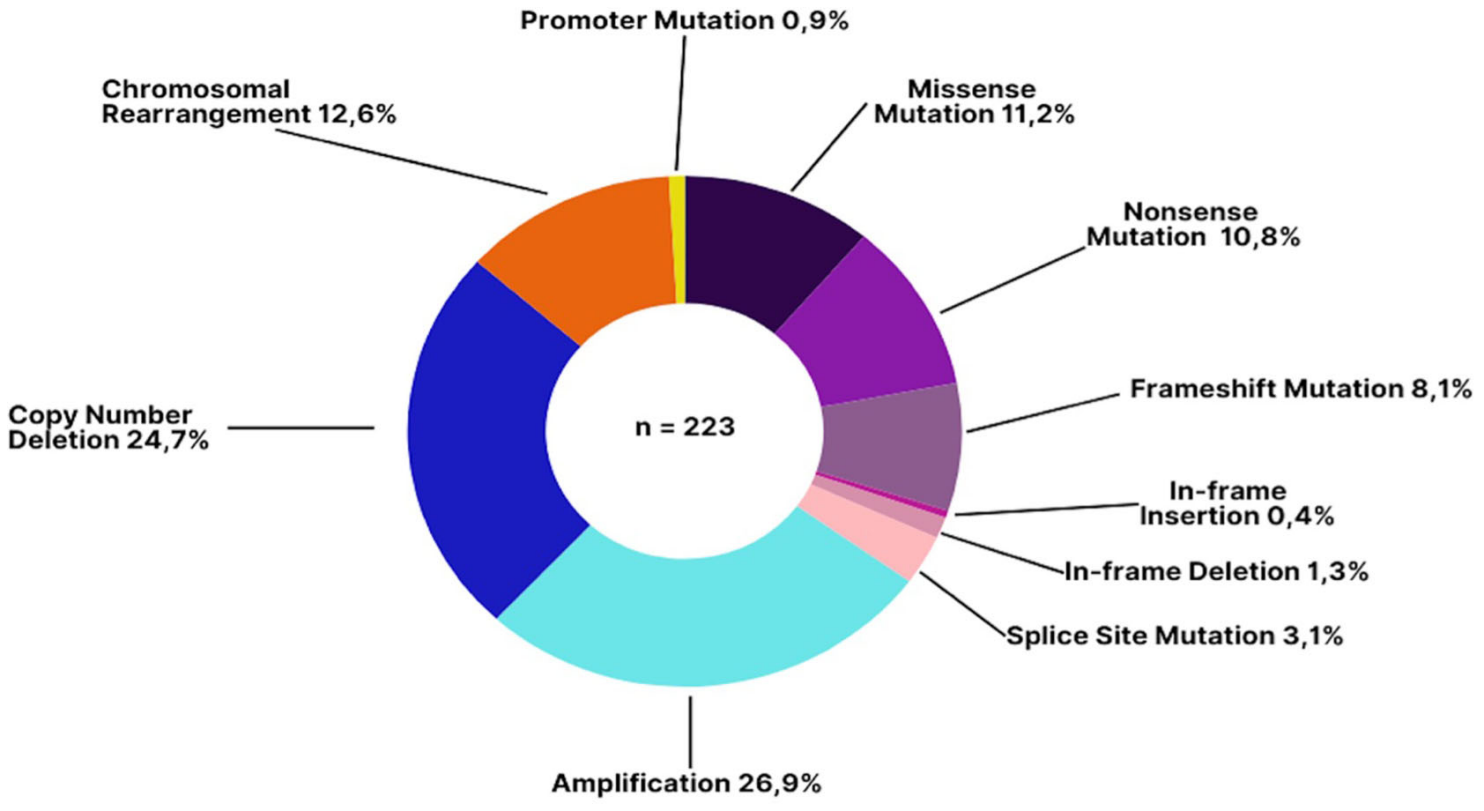
Types of genomic alterations detected in patients with sarcoma. The most common alteration types were amplifications (26.9%) and copy number deletions (24.7%), which are frequently implicated in oncogene activation and tumor suppressor loss, respectively. Chromosomal rearrangements (12.6%)—including translocations such as *EWSR1 fusions*—can have diagnostic utility in specific sarcoma subtypes. Missense (11.2%) and nonsense mutations (10.8%) often result in aberrant or truncated proteins, while frameshift mutations (8.1%) can dramatically alter protein function.

Copy number amplifications were the most prevalent mutation type (26.9%), followed by copy number deletions (24.7%) (**Figure 2**). Chromosomal rearrangements were also notably present, particularly involving the *EWSR1* gene in cases of Ewing’s sarcoma which is also a disease defining gene alteration.

**Figure 3:**
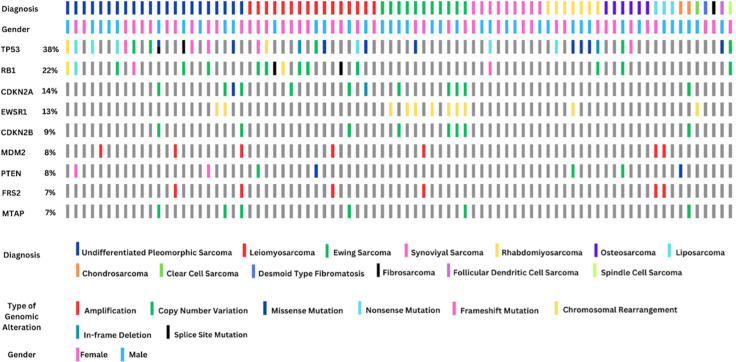
Genomic alteration summary of the most frequently altered genes across 81 sarcoma patients stratified by diagnosis, gender, and mutation type. The top row indicates histologic subtype, with undifferentiated pleomorphic sarcoma, leiomyosarcoma, and Ewing sarcoma being the most represented. Gender is shown in the second row. The remaining rows highlight alterations in the top 10 mutated genes, including TP53 (38%), RB1 (22%), and CDKN2A (14%). Mutations are color-coded by type (e.g., amplifications, copy number variations, missense, and chromosomal rearrangements). Notably, EWSR1 rearrangements (13%) serve as a diagnostic hallmark for Ewing sarcoma, while TP53 and RB1 mutations are common across several subtypes and may be prognostically relevant.

The types of genomic alterations detected in the most frequent genes are summarized in **Figure 3**. From a total of 31 *TP53* alterations detected, 63.2% were loss of function mutations. Among the 18 patients with mutations in *RB1*, 61.1% had copy number variations. Of the 12 patients with alterations in *CDKN2A*, 83.3% had copy number variations.

All genomic alterations involving the *EWSR1* gene were observed to be chromosomal rearrangements. Among the 11 *EWSR1*-related translocations identified, seven were detected in patients diagnosed with Ewing sarcoma, while two were found in UPS, one in rhabdomyosarcoma, and one in a patient diagnosed with clear cell sarcoma. In 42% of the 11 patients histopathologically diagnosed with Ewing sarcoma, no *EWSR1*-related mutation was observed. A total of eight patients exhibited alterations in the *CDKN2B* gene, all of which were copy number losses. Notably, 50% of these patients were diagnosed with Ewing sarcoma. All seven *MDM2* mutations identified were found to be copy number amplifications. Alterations in the *MDM2* gene were detected in 66% of patients diagnosed with liposarcoma. Additionally, *FRS2* amplification was observed in six patients, all of whom also had *MDM2* amplification.

Microsatellite instability (MSI) analysis was performed in 79 patients, all of whom were classified as microsatellite stable (MSS), indicating a low likelihood of response to immunotherapy. Among these patients, the TMB ranged from 0 to 12.1 mut/Mb, with a mean TMB of 2.56 ± 2.31 mut/Mb. The analysis of TMB highlighted potential high mutation burdens in specific cases, notably in a UPS patient with a TMB of 12.1 mut/Mb, suggesting eligibility for immunotherapy.

### Actionability

18 patients (22.2%) had an actionable mutation and were recommended therapeutic option by NGS testing reports. Only 10 patients were able to be treated with NGS-targeted therapy. No statistically significant differences were observed in overall mutation frequencies or in access to targeted therapies when stratified by histology, sex, or age group (p > 0.05).

Three patients had diagnosis-changing results in NGS. First patient’s histopathological diagnosis was UPS, according to the current WHO classification (2), tumors with *CIC-DUX4* fusion are defined as CIC-rearranged sarcomas. Another example of a case where the pathological diagnosis contradicted the NGS profile was a patient with a histopathological diagnosis of UPS, in whom an *EWSR1-ERG* fusion was detected. Based on this finding, we concluded that the patient’s primary diagnosis was Ewing sarcoma. Third patient, whose histopathological diagnosis was defined as UPS but was found to have an *EWSR1-ATF1* fusion, was ultimately determined to have a primary diagnosis of clear cell sarcoma. There was one instance in which the NGS-related findings were diagnosis-modifying. One patient diagnosed with rhabdomyosarcoma harbored *PAX3-FOXO1* fusion mutation, which refined the diagnosis as alveolar rhabdomyosarcoma.

Two patients were identified with significant germline mutations. One patient with epithelioid fibrosarcoma had a germline *BLM* mutation, and another with UPS possessed *TP53* and *ATM* mutations. These patients were further investigated for family risk assessment and were provided genetic counseling. The patient with germline *BLM* (p.W567 nonsense) mutation was diagnosed with Bloom syndrome and the patient with *TP53 (p.R337C* missense*)* and *ATM (p.R1466* missense*)* mutations was diagnosed with Li-Fraumeni Syndrome (LFS). The molecular profiling had been life-changing for two patients.

## Discussion

This study represents the most extensive investigation of unresectable sarcoma patients using comprehensive genomic profiling in Turkey, contributing significantly to the global understanding of sarcoma genomics. Sarcomas are characterized by significant genetic heterogeneity, necessitating precision medicine approaches for improved patient management. Our findings align with prior studies demonstrating that the vast majority of sarcomas harbor detectable genomic alterations, with *TP53*, *RB1*, and *CDKN2A* being the most frequently mutated genes (12). These alterations highlight the importance of cell cycle dysregulation in sarcomagenesis and may serve as potential therapeutic targets. Cole et al. also, in their analysis of 133 sarcoma patients using NGS, reported that the most common mutations were associated with cell cycle regulation (13).

The detection of genomic alterations in 90.1% of our cohort is consistent with previous international studies, which report a similarly high mutational diversity in sarcoma (12–16). However, only 22.2% of these alterations were deemed actionable with currently available FDA-approved therapies, underscoring the ongoing challenge of translating genomic findings into effective treatments, the gap between genomic discoveries and their clinical implementation. The presence of actionable mutations in a subset of patients suggests that precision oncology could play an increasingly critical role in sarcoma management, particularly for patients with limited treatment option (8). Still, NGS profiling had an impact on three patients’ diagnosis and potential prognostic trajectory and been life-changing for 2 patients who were diagnosed with familial syndromes after molecular profiling results.

Among the 81 patients in our study, 8 (9.9%) had no detectable genomic alterations, aligning with findings from Gusho et al. (10.3%) and other studies reporting at least one alteration in over 90% of cases (14–16). The predominant mutations in *TP53* (38%), *RB1* (22%), and *CDKN2A* (14%) were consistent with previous reports (16, 17). Additionally, *PTEN* and *MDM2* mutations were identified as prognostic markers, where *PTEN* alterations were linked to reduced survival in STS (18). *PTEN* mutation was detected in 8% of cases, with loss-of-function mutations identified in 4 patients and copy number loss mutations in 3 patients. Notably, *MDM2* and *FRS2* co-occurrence (85.7%) was frequently observed, particularly in well-differentiated and dedifferentiated liposarcomas, where it serves as a key diagnostic marker (19, 20). Another notable genetic alteration identified through NGS was the *HMGA2-DHX9* fusion. The *HMGA2* mutation, previously described in certain sarcomas, particularly fibrosarcomas, has been associated with treatment resistance (21). To date, no cases of *HMGA2-DHX9* fusion have been reported in sarcoma patients in the literature. Although its clinical significance remains unclear, the mutation is reported in a patient diagnosed with liposarcoma for the first time.

In our study, only 18 patients (22.2%) had actionable mutations with available FDA-approved targeted therapies. The frequency was found to be lower than the previous studies (8). In a recent large-scale analysis, clinically targetable mutations were identified in 31.7% of 7,494 sarcoma cases, where 81% of the cohort had STS (10). Differences in STS (75.3% in our study) distribution may contribute to this variation, as STS is known to harbor more targetable mutations and have broader therapeutic options. Some patients had multiple treatment options, resulting in a total of 34 targeted therapy recommendations. Given that sarcomas are rare and associated with poor prognosis, with survival estimates of 30% at two years (22), the identification of 34 treatment options through NGS in 81 patients underscores the significant role of precision medicine in clinical decision-making for this challenging malignancy. While 22.2% of patients in our cohort had actionable mutations, only 10 were able to receive targeted therapies. This gap underscores key challenges in translating molecular findings into clinical benefit. Barriers include limited access to approved targeted therapies for rare cancers such as sarcomas and lack of reimbursement for off-label use. Additionally, the absence of matched clinical trials and uncertainty among clinicians about the utility of targeted agents in off-label contexts may further limit implementation. Addressing these barriers is essential to fully harness the clinical potential of precision oncology in real-world settings.

Microsatellite instability analysis confirmed that all 79 patients with available data were MSS, consistent with the literature, which reports MSI in less than 1% of sarcomas (23). Consistent with large-scale studies reporting low TMB levels in sarcomas 10, our cohort exhibited a mean TMB of 2.56 ± 2.31 muts/megabase, further highlighting the limited benefit of immunotherapy in this population (24). Given the prevalence of translocation events—associated with relatively low genomic instability—in sarcomas, the observed low TMB levels were not unexpected.

This study underscores the importance of RNA-based NGS in detecting fusion-driven sarcomas, aiding in diagnostic accuracy and guiding treatment. For example, one patient was diagnosed with CIC-rearranged sarcoma based on the detection of a CIC-DUX4 fusion, a rare entity associated with poor prognosis and chemoresistance. Another case involved an EWSR1-ERG fusion in a patient initially diagnosed with UPS, ultimately leading to reclassification as Ewing sarcoma. In our cohort, the integration of genomic data led to a reclassification of initial histopathological diagnosis in four patients. These findings emphasize the diagnostic power of NGS, particularly in cases with ambiguous or overlapping histologic features (12, 25, 26). Such reclassifications can significantly alter clinical management, affecting both treatment strategy and prognosis. In a study done by Atiq et al. (27), a new amplicon-based targeted NGS assay was tested on the first 652 patients and the results also helped in changing or confirming diagnosis as well as identifying novel fusions. These findings support the routine inclusion of molecular profiling in the diagnostic workflow for challenging sarcoma cases.

Furthermore, the detection of significant germline mutations within the cohort suggests that NGS can play a valuable role in individual and familial risk management, providing essential information for genetic counseling and decision-making in cases suspected of hereditary sarcoma syndromes. Germline mutations were detected in two patients included in our study. One of the patients, diagnosed with Bloom syndrome, was enrolled in an intensive surveillance program for other potential cancers and was advised to undergo familial screening. In the other patient, a germline TP53 p.R337C missense mutation and a germline ATM p.R1466 missense mutation were identified. The patient was diagnosed with LFS, and due to the heterozygous ATM mutation, familial screening was recommended for both ataxia-telangiectasia and LFS, alongside cancer surveillance.

Germline mutations and associated hereditary syndromes are known to be more frequently detected in children and adolescents, with reported rates ranging from 4.6% to 10% (28). In our study, germline mutations associated with cancer predisposition were identified in 2.5% of cases, and the affected patients, aged 31 and 39, were relatively young. Approximately 10% of osteosarcomas are associated with hereditary cancer syndromes (29), and the increased familial risk of STS are shown with hereditary retinoblastoma, LFS, familial adenomatous polyposis, neurofibromatosis, tuberous sclerosis and Werner syndrome (30). STS should be evaluated clinically for personal history to be assessed for the possibility of cancer predisposition syndromes. Considering the potential benefits of early detection and treatment of accompanying findings, as well as the advantages of family screening for the healthcare system, evaluating germline mutations through NGS testing in young and middle-aged sarcoma patients may be a reasonable approach.

Despite its potential, NGS application must be tailored to specific sarcoma subtypes and clinical scenarios. Considering the rarity and unfavorable prognosis of sarcomas, integrating genomic profiling into multidisciplinary tumor boards may optimize clinical decision-making. While targeted and immunotherapy options remain limited, NGS serves as a valuable tool for patients with actionable mutations, refractory disease, or hereditary risk factors. Another emerging area of interest in precision oncology is the integration of molecular profiling with imaging characteristics, known as radiogenomics. This approach seeks to correlate imaging features (e.g., from MRI or CT) with underlying genomic alterations, potentially enabling non-invasive tumor characterization. In a study by Crombe et al. (31), underscoring the integration of radiomic and genomic data in STS, the tumors that appeared to evolve most rapidly on imaging scans not only enhanced proliferative activity but also exhibited immune response suppression. In sarcomas, radiogenomic analysis may help understand mutation status, guide personalized treatment, and monitor disease progression.

Limitations of this study include its retrospective design, relatively small cohort size, and the difficulty of capturing long-term treatment outcomes—particularly among international and second-opinion patients. Since the study spans cases diagnosed between 2017 and 2023, the evolving landscape of NGS technologies and the timing of regulatory approvals for targeted therapies may have influenced treatment decisions, especially in earlier cases where fewer therapeutic options were available. Another limitation is that genomic analysis was performed on the initial diagnostic biopsy or resected specimens, which may not reflect resistance mutations that developed under treatment pressure. Moreover, clinical outcome data for patients who received NGS-guided therapies were not consistently available, as many were externally referred and their follow-up occurred outside our institution. This limited our ability to assess the real-world impact of matched therapies. In addition, sequencing was performed without matched normal tissue, which restricts the ability to definitively distinguish somatic from germline variants. While tumor-only sequencing is widely accepted in clinical oncology, matched normal controls could enhance the precision of variant classification, particularly in patients with suspected hereditary cancer syndromes. Furthermore, in tumor-only sequencing, certain features can suggest possible germline origin. These include variants with high variant allele frequency (VAF >50%), pathogenic alterations in well-known hereditary cancer predisposition genes (e.g., BRCA1/2, TP53, ATM, CHEK2), recurrent detection of the same variant across multiple tumor samples in the same patient, and copy number patterns consistent with heterozygous germline deletions. Clinical context, such as young age at diagnosis or a strong family history of cancer, may further strengthen germline suspicion. However, these findings remain presumptive, and definitive classification always requires confirmation by independent germline testing and genetic counseling. Finally, a methodological limitation of this study is the use of multiple NGS platforms with varying gene panel sizes, sequencing depth, and bioinformatics pipelines. These differences may have affected the detection rates of certain alterations and limited the comparability of metrics such as TMB and MSI. Taken together, these factors highlight the broader challenges of implementing genomically guided therapy in sarcomas, where tumor evolution and clonal selection may result in molecular heterogeneity over time. Future studies incorporating dynamic tools such as serial biopsies or liquid biopsies may better capture resistance mechanisms and inform real-time treatment decisions.

## Conclusions

Our study highlights the need to implement genetic information from molecular profiling with clinical insights to optimize the benefits of personalized medicine in sarcoma treatment. Although actionable mutations remain limited, NGS provides critical insights into tumor biology, refines diagnoses, and aids in treatment decision. This research sets the stage for a more targeted method of managing sarcomas, integrating genetic data with clinical practice to improve patient care.

## Data Availability

The data that support the findings of this study are available on request from the corresponding author ES.
